# Towards neonatal mortality risk classification: A data-driven approach using neonatal, maternal, and social factors

**DOI:** 10.1016/j.imu.2020.100398

**Published:** 2020

**Authors:** Carlos Eduardo Beluzo, Everton Silva, Luciana Correia Alves, Rodrigo Campos Bresan, Natália Martins Arruda, Ricardo Sovat, Tiago Carvalho

**Affiliations:** a Federal Institute of São Paulo, Campinas, SP, Brazil; b Department of Demography, University of Campinas (UNICAMP), Brazil

**Keywords:** Infant mortality, Data-driven models, Demographic features, Public health, Understandable model

## Abstract

Infant mortality is an important health measure in a population as a crude indicator of the poverty and socioeconomic level. It also shows the availability and quality of health services and medical technology in a specific region. Although improvements have been observed in the last decades, the implementation of actions to reduce infant mortality is still a concern in many countries. To address such an important problem, this paper proposes a new support decision approach to classify newborns according to their neonatal mortality risk. Using features related to mother, newborn, and socio-demographic, we model the problem using a data-driven classification model able to provide the probability of a newborn dying until $28^{th}$ days of life. More than a theoretical study, decision support tools as the one proposed here is relevant in countries in development as Brazil, because it aims at identifying risky neonates that may die to raise the attention of medical practitioners so that they can work harder to reduce the overall neonatal mortality. Overcoming an AUC of 96%, the proposed method is able to provide not just the probability of death risk but also an explicable interpretation of most important features for model decision, which is paramount in public health applications. Furthermore, we provide an extensive analysis across different rounds of experiments, including an analysis of pre and post partum features influence over data-driven model. Finally, different from previously conducted studies which rely on databases with less than 100,000 samples, our model takes advantage from a new proposed database, constructed using more than 1,400,000 samples comprising births and deaths extracted from public records in São Paulo-Brazil from 2012 to 2018.

## Highlights

• Proposition of a new data-driven support decision approach for neonatal death risk classification.
• A comparative study to assess efficacy of different types of machine learning classifiers in classification task.
• An analysis of feature importance, by a data-driven perspective.
• A comparative study between models constructed using only pre-partum and post-partum features.
• A qualitative analysis of death risk classification comprising not trivial cases.

## Graphical abstract

**Figure:**
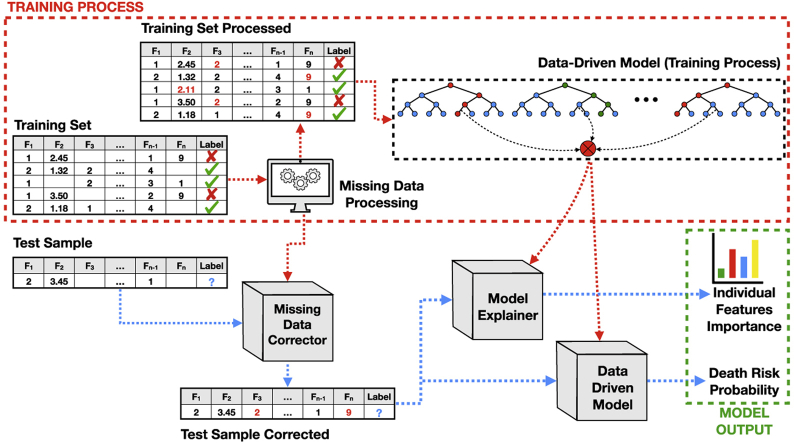

## 1 Introduction

Infant Mortality (IM) is an important health measure in a population and can be considered an indicator of the poverty and socioeconomic level. It also shows the availability and quality of health services and medical technology in a specific region. Comparisons of the Infant Mortality Rate (IMR), which is presented as the deaths of children less than one year old per 1000 alive births, are used in needs assessments and to evaluate the influence of public policies. IM is categorized as neonatal, when the death occurs after postpartum and until 28 days of life; and as post-neonatal, when the death occurs from 29 days of life until one year of life.

**Fig. 1:**
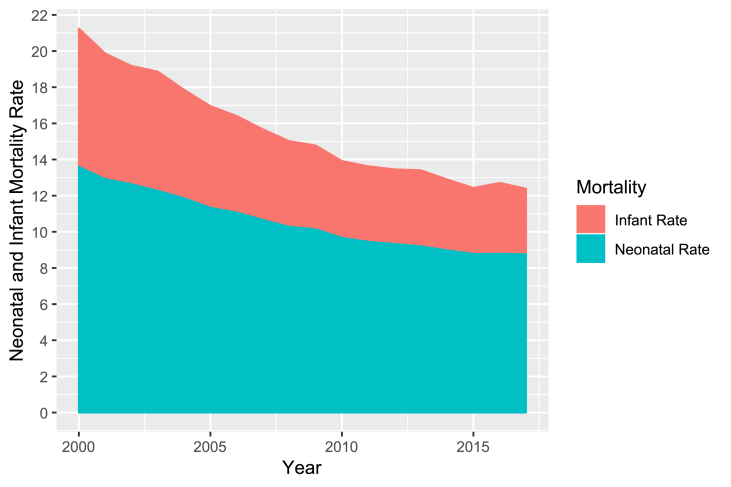
Infant mortality rate and neonatal mortality rate - Brazil from 2000 to 2017. Data source: Datasus 2000–2017.

Neonatal Mortality Rate (NMR) and IMR are very important indicators to measure public health situations and the development level of a country. Actions to reduce infant mortality reflect on these indicators, which can positively influence the landscape of the national public health situation [6]. The neonatal mortality accounts to approximately 60% of the IM in developing countries [33]. This dimension of IM is important because, from the point of view of the World Health Organization (WHO) and the United Nations Children's Fund (UNICEF), the first month of life is the period in which the child is more vulnerable. In Brazil it's been observed an increase in the participation of neonatal mortality in the total of IM cases over more than one decade, as we can see in Fig. 1 [25,34].

Child mortality is a worldwide concern in public health as defined by the United Nations (UN) on the global development goals when setting the reduction of the infant mortality until 2015 as a target. Brazil achieved this Millennium Development Goal, but national rates do not reveal the persistent inequalities remaining between geographic regions and population groups. Regions and populations with lower incomes are at greater risk of infant deaths. In addition to the disparities arising from socioeconomic and geographic factors, infants in the first week of life (early neonatal death) did not reduce satisfactorily and now represent the greatest challenge to the advancement of addressing infant mortality in the country [30].

**Fig. 2:**
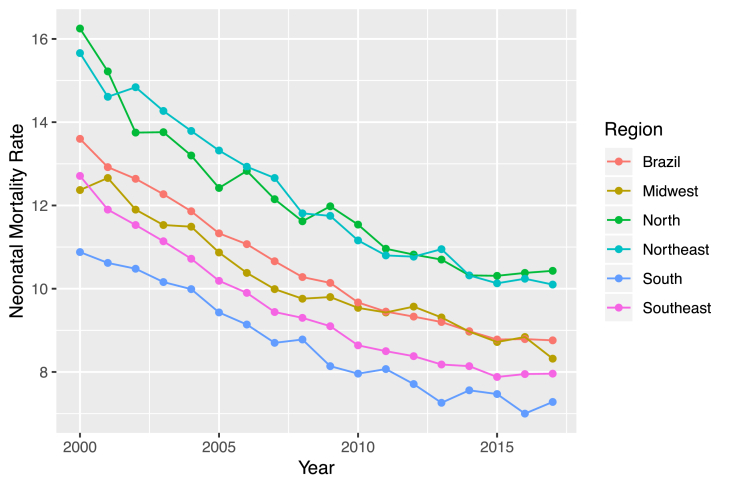
Neonatal Mortality Rate in Brazil and its Macro-regions - from 2000 to 2017. Data Source: DATASUS 2000–2017.

The problem of infant mortality in Brazil has become relevant, since the available data and their respective analysis points to the persistence of disparities between regions, states and populations with different socioeconomic characteristics, despite the constant tendency of general decline [30]. Besides that, evaluating the data from the period of 2015–2017, it can be observed a reverse behavior of the neonatal mortality in Brazil, which after more than 20 years of decline, the NMR started to raise, as illustrated on Fig. 2. Moreover, in Brazil, in 2017, we had a NMR of 9 deaths per 1000 live births, while in developed countries, the NMR is on average 4 neonatal deaths per 1000 live births.

In Brazil, since the enactment of the Federal Constitution of 1988, a large part of the burden of coping with neonatal mortality has been imposed on municipalities, that have adopted a prominent position in the implementation of public health policies [3,14]. São Paulo, the capital city of the state of São Paulo, located in the southeast of Brazil, has the lowest NMR, which was 7.5 per 1000 live births in 2017.

The factors associated with neonatal mortality are deeply articulated and influenced by the maternal and newborn biological characteristics, social conditions and the care provided by the health services [7,20]. In 2003, Mosley and Chen proposed a hierarchical model based on the hypothesis that socioeconomic factors determine behaviors that, in turn, have an impact on a set of biological factors [19]. According to their model, biological factors are those directly responsible for death. The hierarchical model brings great advance to the development of public policies, since information coming from studies that are limited to only a group of risk factors result in inadequate recommendations to assess the deaths among children, as they present a limited vision of the phenomenon.

For the most part, diagnostics are highly dependable on the acquired experience of the professional who performs it, as well as the human intellectual capacity to analyze available data about a certain disease and about the patient's history that is being analyzed. Although being unquestionably necessary, it isn't flawless, specially for the fact of being dependent on human factors, and that is why the professionals have always had technological resources to aid in this task [1].

We have recently observed a great raise in the availability of diagnostic support systems, specially computer systems based on Artificial Intelligence techniques [35]. These systems are indispensable mainly when dealing with a certain amount and availability of data, which can reveal important knowledge, but that are not susceptible to human analysis in a timely manner to assess the problem [28].

In this paper, based on the scientific hypothesis that unusual combinations of different features can lead to neonatal mortality risk, and that together these features can help construct a fully data-driven model to detect neonatal mortality risk, we present a new approach to model neonatal death risk. In contrast to classical statistical models, the proposed approach relies on knowledge learned from an extensive database, comprising more than 1,400,000 records from São Paulo city - Brazil between 2012 and 2018. Furthermore, this kind of study is relevant in countries in development such as Brazil, because it aims to identify risky neonates that may die to raise the attention of medical practitioners so that they can work harder to reduce the overall neonatal mortality.

### 1.1 Machine learning applied to public health and demographic research

Most of the demographic studies in Brazil search for specific factors related to infant and neonatal mortality, based on the use of descriptive analyses like spatial analysis, multiple statistical and logistic parametric regression and, in general, using small datasets.

Nascimento et al. proposed an hierarchical model to analyze a dataset with 264 neonatal deaths while Migoto et al. uses a dataset containing 157,604 live births and 903 early neonatal deaths (up to the sixth day of life). Both works found some strongly related factors to total neonatal mortality and early neonatal mortality. It was observed that neonatal deaths were related mainly to the quality of the prenatal care and direct care labor. These features were measured through some variables such as: number of prenatal consultations, type of labor, professional responsible for the childbirth (doctor on call, obstetrician, nurse or other). In addition, some associations were found regarding the reproductive history of the mother, such as if the mother presented fetal losses in previous pregnancies. They also identify some relation with presence of malformation and to maternal socioeconomic conditions (mother's education, marital status and mother's race) [17,20].

Migoto et al. also points out that maternal age indicates a higher chance of early neonatal death among adolescent mothers and those who were 35 or older when compared to mothers who were 20–34 years old. Regarding the education, the mothers of children who died before completing one week of life studied until they were seven. Moreover, the children of women who had no partner were more likely to die when compared to women who were married [17].

While aforementioned works rely on traditional statistics methods, Machine Learning (ML) approaches start to be highlighted among some international works. Nguyen proposes the use ML of approaches to analyze in-hospital child mortality using as features the final diagnosis, for example, children with meningitis or malnutrition diagnosis were most likely to die. This work had the concern to make models that were capable of detecting the death in the sample since the outcome was a rare event [21].

Pan observed that ML models were capable of identifying more then 150 high risk pregnant women besides the paper-based risk assessment already used in the social services in Illinois [26]. So, the ML methods were capable of improving the efficiency of decision making and improvements in the identification of high-risk pregnancy eligible to receiving specific health services.

The ML approach proposed by Podda et al. aimed at estimating the survival of newborns prematurely and compared ML models with the most common logistic methods in these types of analyzes. Author's methods predict the survival of preterm neonates better than logistic models and, thus, allow a better approach for identifying risks and allowing the improvement of decision quality and identification of risks. They used neural networks and observed that although logistic regression models and other linear models are more easily understood and interpretative, and their results are easily used as risk measures; this ease of interpretation is lost when interactions between variables are present, and in this case, neural networks can take into account interaction between variables and non-linearities with the variable outcome [27].

Hsieh proposed a comparison of ML models with the aim to predict the mortality of patients with unplanned extubation in intensive care units. They observed that even with limited data points (341), they were able to develop a good prediction model. These authors worked with an unbalanced data, with the *Random Forest* model presenting best recall and precision values compared to the others models used in that work (Support *Vector Machine, Artificial Neural Networks, Logistic Regression Model*) [11].

To the best of our knowledge, there are no data-driven models constructed from Brazilian data to analyze neonatal death risk. As well, there are no reported results for this kind of problem using features from Mortality Information System (SIM - Sistema de Informação de Mortalidade) and the National Information System on Live Births (SINASC - Sistema de Informação de Nascidos Vivos). Then, the main contributions of this paper are: (1) proposition of a new data-driven support decision approach for neonatal death risk classification, which combines different state-of-the-art computational methods to solve an open problem, providing a quantitative measure of how neonatal mortality risk can be low or high, which can help doctors as a decision support tool; (2) a comparative study to assess efficacy of different types of ML classifiers in the classification step; (3) an analysis of feature importance, by a data-driven perspective, using the *Shapley Additive Explanations* method (SHAP - SHapley Additive exPlanations), which is essential to improve problem understanding as part of proposed method; (4) a comparative study between models constructed using only pre-partum and post-partum features; (5) a qualitative analysis of death risk classification comprising not trivial cases.

Our method can be considered as a diagnosis tool to support decision in evaluating the risk of a patient suffering neonatal death, once all the necessary features are available before the birth (pre-partum) and a few hours after the birth (post-partum). Therefore, the method can increase the efficacy and the precision in the task to evaluate risks and to provide guidance on the need of post-partum therapy in an initial stage, saving the life of newborns in a last resort, as well as reducing the cost of treatment, considering that it can also prevent the use of unnecessary resources in newborns that are not at risk.

The rest of this work is divided as following: Section 3 presents details of the methodology including classification algorithms description and experiments protocol defined for this paper, as well as the dataset construction process and respective exploratory analysis. Section 4 brings details of performed experiments and respective results, including results of the feature importance method. Finally in Section 5 a discussion of the results is presented, along with the main conclusions and perspectives for future works.

## 2 Dataset construction and exploration

The data-driven model proposed along this paper has been constructed by using records extracted from birth and death systems, SINASC and SIM respectively, from São Paulo city between 2012 and 2018. São Paulo city provides one of the data quality sources in Brazil and the data was collected directly from the Municipal Health Office of São Paulo (SMS - Secretaria Municipal de Sáude de São Paulo).

**Fig. 3:**
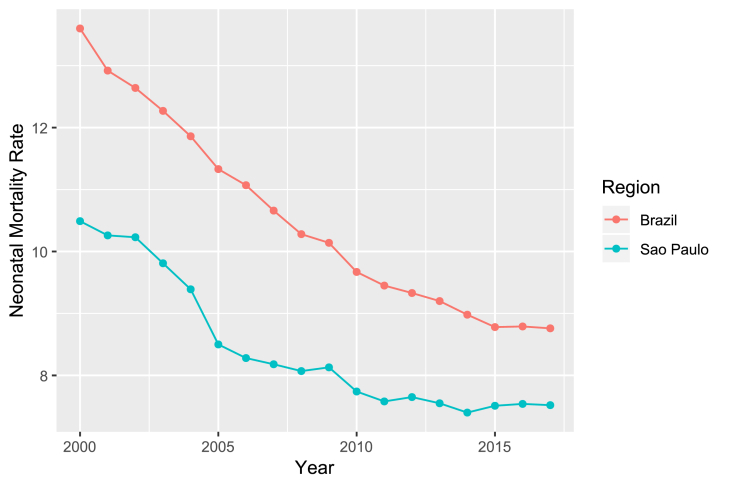
Comparison between Neonatal Mortality Rate of São Paulo city and Brazil from 2000 to 2017. Its important to highlight here that, on this Figure, even on years not covered by this study (before 2012), São Mortality rate is expressive lower than Brazilian rates.

Although São Paulo has the best quality and best levels of neonatal rate when compared to the rest of Brazil, as depicted in Fig. 3, these events occurred in heterogeneous ways with smaller or even lower reductions in the most vulnerable populations, as the reflection of unfavorable life conditions of the population, health care and socioeconomic inequalities [9].

### 2.1 Data Sources

SINASC is fed using the Live Birth Statement (DNV - Declaração de Nascido Vivo). It comprises information about demographic and epidemiological data for the infant, mother, prenatal care and childbirth. On the other hand, SIM has the main goal of supporting the collection, storage and management process of death records in Brazil, and was used to label records where death happened until 28 days of life on SINASC, using DNV, which is a common field in both systems, as an association key. This way, each sample in our final dataset comprises SINASC features and a label (0 or 1) describing if the subject survived, or not, after 28 days of life [15,18,24,32].

DNV is a standard document prepared by the Ministry of Health and mandatory throughout the national territory for the registration of a child's birth. It must be filled in all live births, whatever the circumstances of the birth: hospitals, maternity, emergency, household, public roads, vehicles, etc. Similarly, we have the death certificate (DO - Declaração de Óbito) that is the document used to collect information about mortality and it is used as the basis for the calculation of vital statistics, such as the calculation of the Brazilian neonatal mortality rate.

However, even though filling out the DNV and the DC is mandatory, there is a significant deficiency in data quality due to many situations as losses when sending them from hospitals to the city health offices (which is responsible to report to the MS); fields filled with incorrect values; unknowing information by the person answering it, etc.

Table 1 describes the amount of missing data per feature in our dataset. At least 10% of data samples in SIM/SINASC data-sources present at least one missing/inconsistent feature.

**Table 1:** Clinical characteristics of patients and controls.

	All participants (n=698)		
	AD (n=400)	Control (n=289)	*P*
Female (n, %)	281 (70.25%)	207 (71.63%)	0.70
Male (n, %)	119 (29.75%)	82 (28.37%)	
APOEε4 (+) carrier (n, %)	155 (38.60%)	51 (17.64%)	<0.001
APOEε4 (-) carrier (n, %)	245 (61.40%)	238 (82.36%)	
CLU rs11136000			0.04*
T (n, %)	164 (21.08%)	131 (23.48%)	
C (n, %)	614 (78.92%)	427 (76.52%)	
rs2279590			0.03*
A (n, %)	160 (20.62%)	127 (22.68%)	
G (n, %)	616 (79.38%)	433 (77.32%)	
rs9331888			0.44*
C (n, %)	386 (48.74%)	279 (49.47%)	
G (n, %)	406 (51.26%)	285 (50.53%)	
Age, years (mean ± SD)	80.1± 6.99	71.0±6.29	<0.001
BMI, kg/m2 (mean ± SD)	22.75±3.85	23.99±3.78	<0.001
TG, mmol/L (mean ± SD)	1.37±0.61	1.45±1.05	0.17
HDL-C, mmol/L (mean ± SD)	1.50±0.43	1.54±0.44	0.16
LDL-C, mmol/L (mean ± SD)	3.11±0.82	3.03±0.84	0.20
DWRT score (mean ± SD)	0.31±0.91	7.22±1.26	<0.001

*APOE*, Apolipoprotein E gene; BMI, Body Mass Index; TG, Triglyceride; HDL-C, High-density Lipoprotein Cholesterol; LDL-C, Low-density Lipoprotein Cholesterol; DWRT, Delayed Word Recall Test.

*P value adjusted by age, gender, BMI and *APOE ε4.*

Using SINASC and SIM we constructed a dataset named **SPNeoDeath**, and it comprises 1,427,906 samples, with 23 features (and the target variable). The entire set of features are described on Table 2.

**Table 2:** Dictionary of data - variables of the dataset.

Feature	Description
maternal_age	Mother's age
newborn_weight	birth weight (grams)
cd_apgar1	1-min Apgar score
cd_apgar5	5-min Apgar score
cd_robson_group	Robson group classification
num_cesarean_labors	Number of cesarean deliveries
num_fetal_losses	Fetal losses
num_gestations	Number of previous gestation
num_gestational_weeks	Gestational weeks
num_live_births	Number of live births
num_normal_labors	Number of normal deliveries
tp_birth_place	Birth place code
tp_childbirth_assistance	Childbirth care
tp_fill_form_responsible	Main worker role
tp_labor	Child-birth type (delivery)
tp_maternal_skin_color	Mother race/skin color
tp_marital_status	Marital status
tp_maternal_education_years	Mother's years of schooling
tp_pregnancy_duration	Week of gestation (by ranges)
tp_pregnancy	Type of pregnancy
tp_prenatal_appointments	Prenatal appointments (by range)
tp_presentation_newborn	Newborn presentation type
has_congenital_malformation	Congenital malformation

Data distribution between positive (death) and negative (alive) classes is presented on Table 3.

**Table 3:** Distribution of samples across dataset classes.

	Positive Class **(Death)**	Negative Class **(Alive)**
Total of **Samples**	7928	1,427,906
Dataset **Proportion**	0.55%	99.5%

### 2.2 Dataset description and exploratory analysis

For a better understanding of the **SPNeoDeath** dataset and the delimitation of the problem, this section provides some insights about the dataset by an exploratory analysis. The dataset can be divided in four categories: (a) socioeconomic maternal conditions features; (b) maternal obstetrics features; (c) newborn related features; and (d) previous care related features. Furthermore, some statistics regarding the features values distribution among target classes (deaths or alive) are also presented.

1. **Socioeconomic maternal conditions features:** includes features as age, years of schooling, marital status and race/skin color. According to data distribution, mother's age has a mean value of 28.17 years old, with 75% of the sample concentrated from 15 to 32 years old; 55% of mothers were married or in a stable relationship and 43.34 were single; 58% of the mothers had from 8 to 11 years of education; and 57.83% were white and 40.69 were black or brown.
2. **Maternal obstetrics features:** includes features such as the number of live births, number of previous fetal losses, number of previous pregnancies, number of normal and cesarean labor and type of pregnancy. For all these features, except type of pregnancy, the distribution is presented massively (more than 96% of all samples) with mean of 1 live birth. In relation to fetal losses, most of the sample had between 0 and 1 fetal loss in previous pregnancies. According to dataset distributions, most of the sample had between 0 and 1 previous gestation and previous normal labor. In relation to previous cesarean labor, most of the sample didn't have previous cesarean labor and 97% had a unique pregnancy and 2.74% double pregnancy.
3. **Features related to the newborn:** birth weight, number of pregnancy weeks, 1-min Apgar score, 5-min Apgar score, congenital anomaly and type of presentation of the newborn. Regarding the weight of the newborn, most of the sample was between 2870 and 3500 g with a mean value of 3143 g. Most of the newborns were born between 38–39 weeks, with mean of 38 weeks and minimal value of 15 weeks. In relation to Apgar scores, the majority scored 8 on 1-min Apgar score and 9 at 5-min Apgar score (85% and 93%, respectively), 98% didn't had any congenital malformation and 96.03% had a cephalic presentation.
4. **Features related to previous care:** number of prenatal consultations, labor type, childbirth care and Robson 10-groups classification. Finally, most mothers have been to more than 7 prenatal appointments (78.23%); 56.78% had cesarean labor; 81.83% had childbirth care for the doctor; and 34.52% were in Robson Classification Group 2.

#### 2.2.1 Features values distribution among target classes

From all the features, we selected two from which group previously presented to make some additional analyses using values distribution among death and alive classes.

**Fig. 4:**
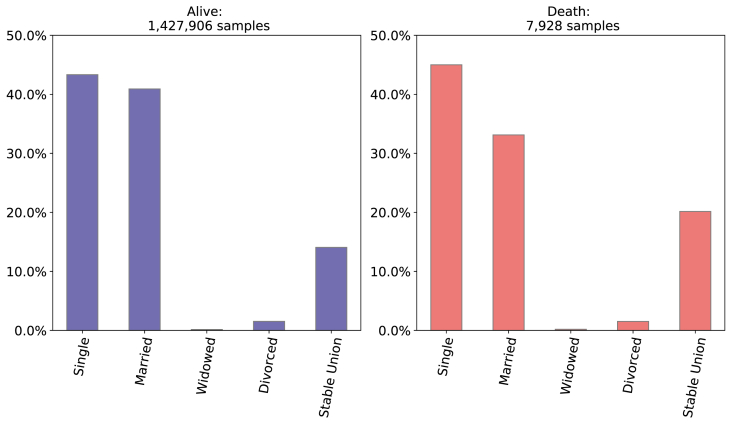
Comparison of Mother's Marital Status between death and alive target variable. Source: SIM, SINASC, 2012– 2018.

**Fig. 5:**
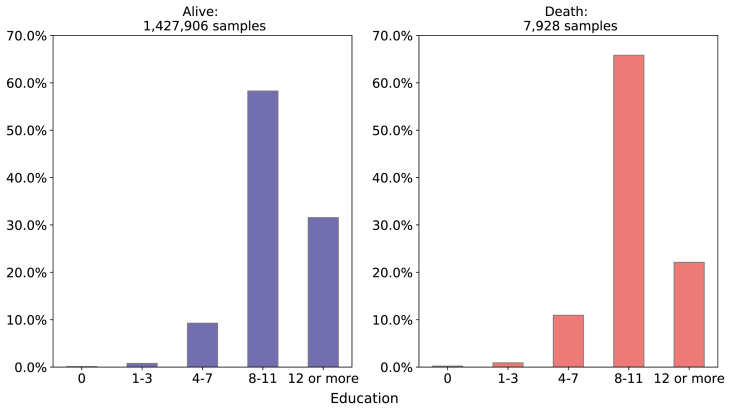
Comparison of Mother's education between death and alive target variable. Source: SIM, SINASC, 2012–2018.

**Related to socioeconomic maternal conditions - Marital Status and Education**:

From all neonatal deaths, 45.00% were single and 53.27% were married or in a stable union and 65.83% had 8–11 years of education and 22.11% had 12 or more years. Observing only those newborn who survived, 43.33% were single and 54.97% were married or in a stable union, and 58.28% had 8–11 years of education and 31.55% had 12 years or more as depicted in Fig. 4, Fig. 5.

**Fig. 6:**
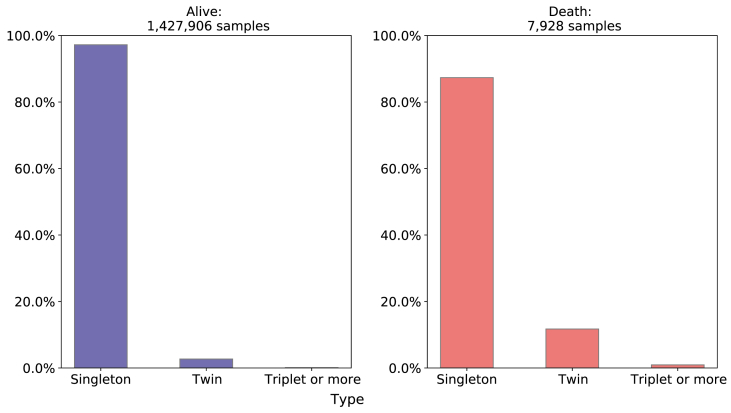
Comparison of Type of pregnancy between death and alive classes. Source: SIM, SINASC, 2012–2018.

**Maternal Obstetrics - Number of previous fetal losses and Type of pregnancy**: Observing Fig. 6, 11.73% of neonatal deaths occurred with double pregnancy and 87% with unique ones and in the alive class, 97.21% were unique pregnancies and only 2.68% were double.

**Fig. 7:**
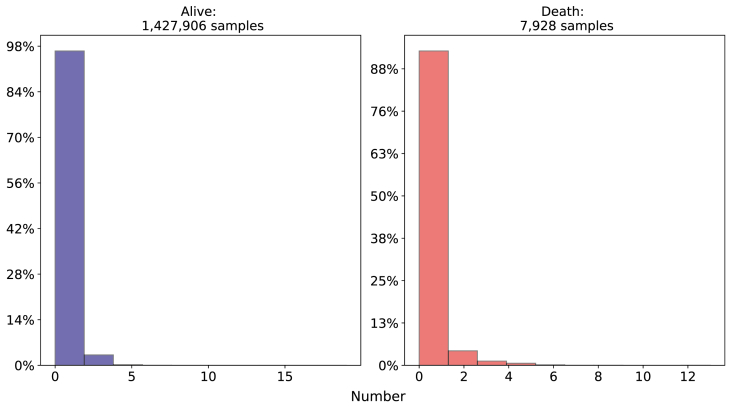
Comparison of previous Fetal Losses between death and alive classes. Source: SIM, SINASC, 2012–2018.

In relation to the historic of fetal losses, the alive class has a concentration of 0–1 fetal losses (almost 98%) and between the newborns who died, the number of mothers who had more than 1 fetal loss before is slightly higher, as we can see on Fig. 7.

**Fig. 8:**
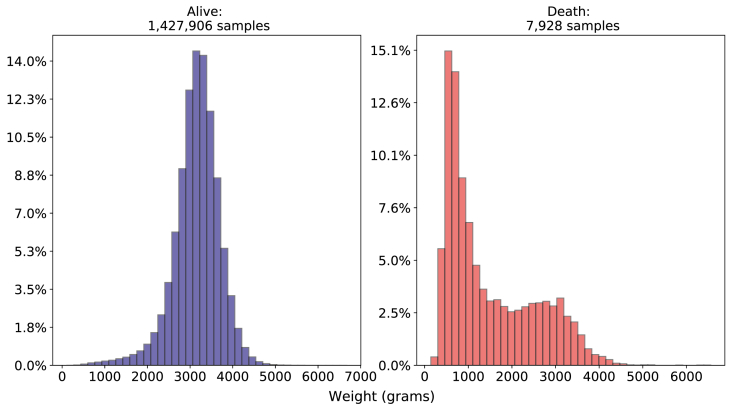
Comparison of birth weight between death and alive classes. Source: SIM, SINASC, 2012–2018.

**Related to the newborn - birth weight and gestational weeks**:

When analyzing birth weight, whose histogram is depicted at Fig. 8, the newborns who died before the 28th day of life (death class) had insufficient weight - with a mean value of 1500 g and most of them concentrated between 660 and 2295 g whereas in the alive class the newborns presented a mean weight value of 3152 g and most of them were concentrated between 2875 and 3490 g.

**Fig. 9:**
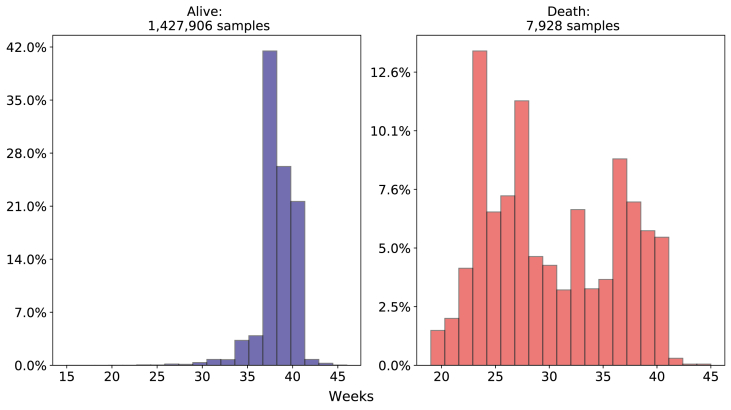
Comparison of Gestational Weeks death and alive classes. Source: SIM, SINASC, 2012–2018.

Regarding gestational weeks, we can see in Fig. 9 considering the death class, most of the sample was born preterm (born before the thirty-sixth week of gestation), with a mean value of 30 weeks and a minimal value of 19 weeks of gestation. In the alive class, most of the newborns were born with a mean of 38 weeks of gestation and most part of the newborn concentrated between 38 and 39 weeks of gestation.

**Fig. 10:**
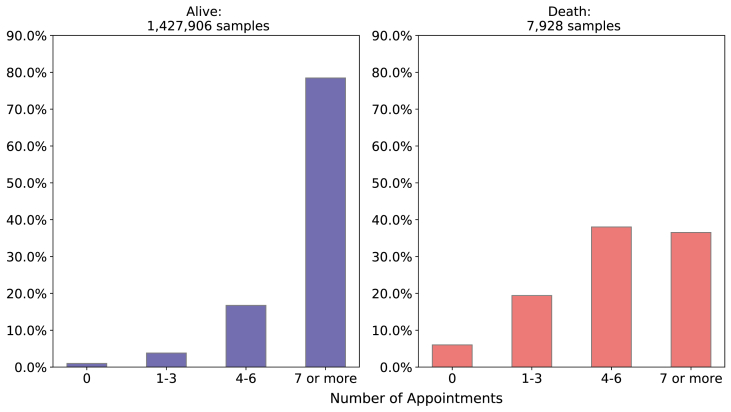
Comparison of Prenatal consultation between death and alive target variable. Source: SIM, SINASC, 2012–2018.

**Related to previous care - number of prenatal appointments and type of labor**: Related to the number of prenatal consultations of newborns who died, 38.02% went to 4 to 6 consultations, 36.54% went more than 7 times and almost 19.41% went only 1 to 3 times. Observing only those newborn who survived, we have 78.46% of mothers went to more than 7 consultations and only 16.73% of those mothers went to 4 to 6 and only 3.8% 1 to 3 times, as depicted in Fig. 10.

**Fig. 11:**
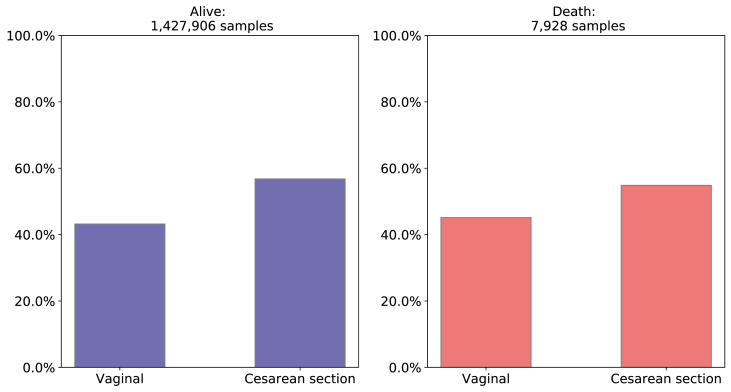
Comparison of Type of Labor between death and alive classes. Source: SIM, SINASC, 2012–2018.

In relation to type of labor, there is no significant difference between alive and death class, that is, 56.79% and 54.85% had a cesarean labor in alive class and death class respectively as Fig. 11 shows.

## 3 Proposed method

**Fig. 12:**
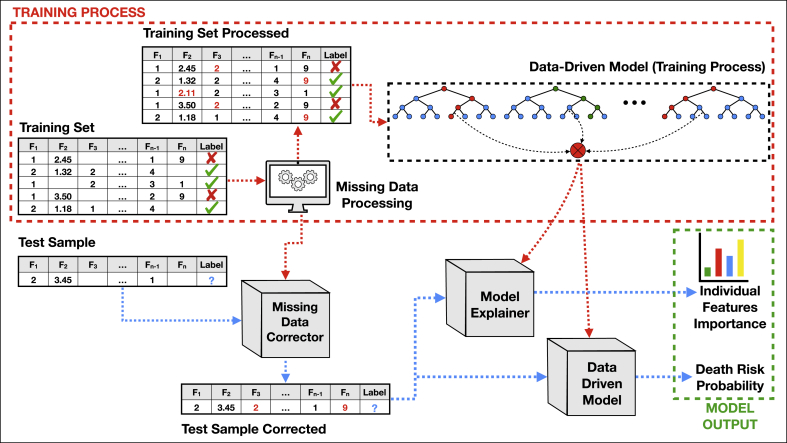
Proposed method overview: the method proposed here is composed of three main sequential steps which involves a missing data processing, followed by a data-driven model classification and interpretation.

The method proposed in this paper,^1^ depicted in Fig. 12, follows three main steps:

1. **Missing Data Processing:** the first step of the proposed method consists in treating input data to deal with data gaps (very common for this kind of data). This process is necessary to allow our data-driven model to learn relationships between different types of features (categorical, ordinal and continuous).
2. Data-driven Model Construction: the second step consists in classifying features provided in step 1 using a supervised learning method. Based on empirical tests, detailed in Section 4.2, our method takes advantage of Extreme Gradient Boosting to perform this task.
3. **Model Explanability:** the last step of the proposed method consists in explaining data-driven model outputs, to make results understandable for human beings. This is one of the core requirements in solutions developed for public health once, its applicability as support decision tools depends on this factor. Then, we applied the SHAP method to this purpose.

### 3.1 Missing data processing

As mentioned above, the first step of the proposed method consists in constructing feature vectors properly, treating missing data.

In the context of Brazilian public health data, occurrence of missing or inconsistent data is usual and it mostly happens due to the incorrect filling of handwritten forms. This kind of data inconsistency could prevent the proposed method to be applied in a real world scenario, once some of the input features for model decision could be missing.

In order to take care of this problem and inspired by demographic studies, our method applies two different approaches: for each continuous feature, we calculate an average value in the training set and use this value to replace missing data in a sample. When dealing with categorical and ordinal features, missing values are filled using the most frequent value for this feature in training sets [16,22].

### 3.2 Data-driven model for death risk classification

Once data is processed, taking care of missing data, the next step of the proposed method is constructing a data-driven classification model able to learn directly from data, the patterns that allow the proposed method to classify a new sample (subject) according to his death risk until 28 days after being born.

The proposed method uses a scalable end-to-end tree boosting method called *Extreme Gradient Boosting* (XGBoost - eXtreme Gradient Boosting), which has as the major advantages regarding the capacity to handle massive volumes of data without the necessity of powerful hardware [5].

In a nutshell, XGBoost is an improved version of Gradient Boosting, and implements a series of improvements to work more efficiently [8]. The main characteristics which lead the proposed method to chose XGBoost, besides the fact that it activates the best results in our data as presented in Section 4, are:

• **regularization**: the XGBoost algorithm uses an specific regularization strategy through both L1 and L2 regularization, which penalizes the complex models and helps to avoid overfitting. In the proposed approach, this is very useful to avoid overfitting models to a high accuracy in no death risk class, since the number of samples in death risk class is much smaller than the no death risk class;
• **handling sparse data**: the XGBoost algorithm incorporates a sparsity-aware split finding algorithm to handle different types of sparsity patterns in the data, as the ones generated by dummy variables and data processing. Specifically in the proposed approach, this kind of feature is very useful since we implement a previous step which processes the data to correct missing and inconsistent data;
• **weighted quantile sketch**: one of the characteristics of most tree based methods is that they rely on the idea that splits are performed using points of equal weights (using quantile sketch algorithm). However, there are no guarantees that data is weighted equally, and handling this kind of scenario is essential when dealing with unbalanced data. XGBoost has a distributed weighted quantile sketch algorithm to effectively handle this kind of limitation;
• **block structure for parallel learning**: to allow a faster training process of the algorithm, even in a dataset comprising more de 1,400,00 samples, XGBoost can make use of multiple cores on the CPU and GPU. Specifically, XGBoost is designed using a block structure, where data is sorted and stored in in-memory units called blocks. Then, data layout can be reused by subsequent iterations, instead of computing it again;

### 3.3 Model explainer

Interpretability of ML models is a concern across different fields in computer science and applied sciences. For public health and demography, this kind of characteristic is specially important. An expert which holds its recommendation using as help a ML model, needs to explain and justify the presented conclusions. In this sense, besides the results of the proposed method execution, on this paper it was also applied the SHAP method, to measure features’ importance and provide a better interpretation of results.

The SHAP method belongs to *additive feature attribution methods*, which can be simplified by linear function of features. This method tries to come up with a linear regression model for each data point. It replaces each feature ($x_{i}$) with a binary variable ($z{'}_{i}$) that represents whether $x_{i}$ it's present or not in the model given by:

(1) $$g\left(z'\right)=\varphi_{0}+\sum_{i=1}^{M}\varphi_{i}z{'}_{i}$$

where g(z') is a local surrogate model of the original linear model f(x) and $\varphi_{i}$ is how much the presence of the feature *i* contributes to the final output, which helps to interpret the original model [13].

Formally, a SHAP value measures the influence of a feature *i* to the output $f_{x}$ produced by a ML model by including the feature *i* for all the combinations of features other than *i* defined by:

(2) $$\varphi_{i}=\sum_{S\subseteq N\setminus \left\{i\right\}}\frac{\left|S\right|!\left(M-\left|S\right|-1\right)!}{M!}\left[f_{x}(S\cup \left\{i\right\}\right)-f_{x}\left(S\right)]$$

where *S* is the subset of features from all features *N* except for feature *i*, $\frac{\left|S\right|!\left(M-\left|S\right|-1\right)!}{M!}$ is the weighting factor counting the number of permutations of the subset *S*, $f_{x}\left(S\cup \left\{i\right\}\right)$ is the actual output model given all features N (including *i*) and $f_{x}\left(S\right)$ is the expected output given the features subset *S*.

In general, the importance is calculated from the reduction of the square error, that is, if this feature was selected in a mode division, in the tree build, and the square error decreased in relation to all the trees. In this paper, the feature importance was calculated by using a Python pre-built library H2O [10].

## 4 Experiments and results

This section presents the main experiments performed along proposed method evaluation.

### 4.1 Computational environment setup

The proposed method has been implemented with the *Python* programming language (version 3.6), along with the *Scikit-Learn* (version 0.21.2), *H2O* (version 3.24.0.4), *XGBoost* (version 0.90), *Pandas* (version 0.24.2) and *MatplotLib* (version 3.1) libraries. All the experiments have been performed in a computer having 40 CPU cores, 4 GPU TitanX 12 GB, 120 GB of RAM and 8 TB of storage, running Ubuntu 18.04 (64 bits).

### 4.2 Experiment #1: evaluating different classifiers in a sub-set of SPNeoDeath

The first experiment conducted is focused on evaluating how the proposed method had been designed, and support our decision by choosing XGBoost as our classification method.

To conduct a fair comparison between methods we first need to address the massive difference in the number of negative and positive (alive and death respectively) classes samples, as described in Section 2.2. This is because the process of training a ML classifier using all the samples inevitably produces an overfitted model.

To handle this kind of problem, we used re-sampling to randomly under-sample the majority class. Our process consists in randomly selecting 70% of samples in positive class (Death) from SPNeoDeath, resulting in 5549 samples. Then we also randomly selected 5549 samples of negative class (Alive), resulting in a sub-set composed, in total, by 11,098 samples. From here, this sub-set of samples will be named **S1-SPNeoDeath**.

We did compare XGBoost against two literature methods for classification: classical *Logistic Regression* and *Random Forest*. At this point, to provide a fair comparison between the methods, all of them have been tested using default parameters provided by Scikit-Learn library.

**Fig. 13:**
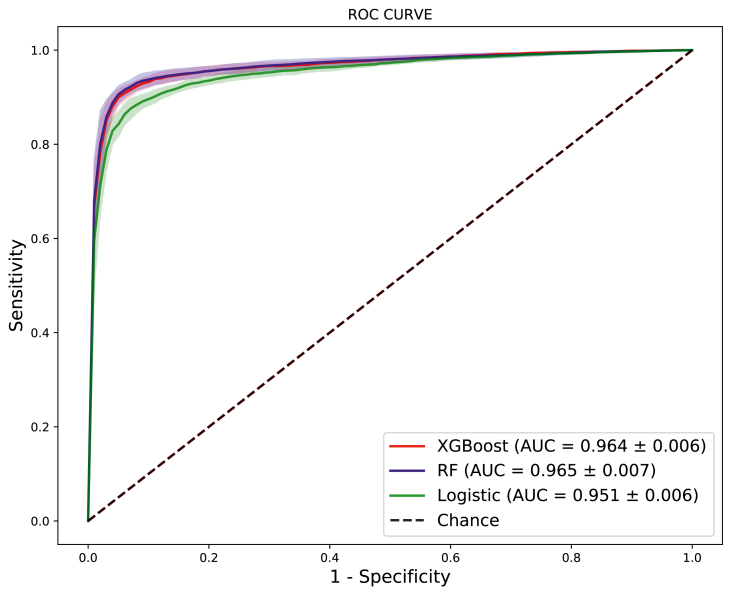
Results using 10-folds cross validation experiment for 3 for proposed method comparing 3 different algorithms for classification task.

Given that, on this experiment, we are evaluating reduced number of samples, we decided to use 10-folds cross-validation protocol. Results are presented using ROC-Curves, the Area Under the Curve (AUC) metric, as depicted on Fig. 13. Each curve represents the average of 10-folds curve for that method, and the shade around the curve inform its standard deviation across all 10-folds.

All the classifiers evaluated present a very similar AUC, with XGBoost equals to 0.964, *Random Forest* equals to 0.965 and *Logistic Regression* equals to 0.951. Standard deviation presented were 0.006, 0.007, and 0.006 for XGBoost, *Random Forest* and *Logistic Regression*, respectively.

Besides computational advantages of XGBoost, as discussed in Section 3.2, we also applied an statistic significance evaluation based on McNemar's Test, which measures the agreement of two classifiers in incorrect classified samples [12]. When comparing XGBoost against *Random Forest*, results obtained were an statistic of 12.461 and a p-value equals to 0.000, considering 5% of significance. This allow us to reject null hypothesis, which means there is a statistical significance between both classifiers. Comparing XGBoost against *Logistic Regression*, obtained results where 18.921 and 0.000 for McNemar's statistics and p-value respectively. Again, there is an statistical difference between classifiers answers.

We also included in Table 4 performance results using Accuracy, Specificity and Sensitivity.

**Table 4:** Performance metrics for each algorithm.

S/N	Algorithm	Accuracy	Sensitivity	Specificity	AUC
1	XGBoost	92%	91%	94%	0.964
2	Random Forest	93%	92%	94%	0.965
3	Logistic Regression	90%	88%	93%	0.951

#### 4.2.1 XGBoost fine-tuning

Once we decided to use XGBoost as the classification algorithm in the proposed method, we then performed a fine-tuning process to estimate better parameters for the classifier using our features. This fine-tuning process has been conducted using a grid-search approach over S1-SPNeoDeath. The reason we decided to conduct grid-search using S1-SPNeoDeath instead of SPNeoDeath is because the computational cost to perform a grid-search procedure in a dataset containing more than 1.4 M samples is too expensive.

Hyper-parameters on grid search have been evaluated considering the following parameter: min_child_weight (MCW), gamma, subsample, colsample_bytree (CSBT),max_depth (MAXD), scale_pos_weight (SPW). Table 5 presents evaluated values for each parameter.

**Table 5:** Evaluated values on grid-search for each XGBoost hyper-parameter.

MCW	Gamma	Subsample	CSBT	MAXD	SPW
[1,5,10]	[0.5, 1, 1.5]	[0.5, 0.8, 1.0]	[0.5, 0.8, 1.0]	[2,4,6]	[1,5,10]

Therefore, the best hyper-parameters founded are presented in Table 6.

**Table 6:** XGBoost hyper-parameters after grid-search.

MCW	Gamma	Subsample	CSBT	MAXD	SPW
5	0.5	1.0	0.5	2	1

### 4.3 Experiment #2: evaluating SPNeoDeath

Once XGBoost has been validated as the most appropriate algorithm for the classification step of the proposed method, including an additional grid-search step, in the second experiment we evaluated SPNeoDeath entirely. This evaluation was conducted in two different variations described below.

#### 4.3.1 Variation #1: balanced dataset for training

**Fig. 14:**
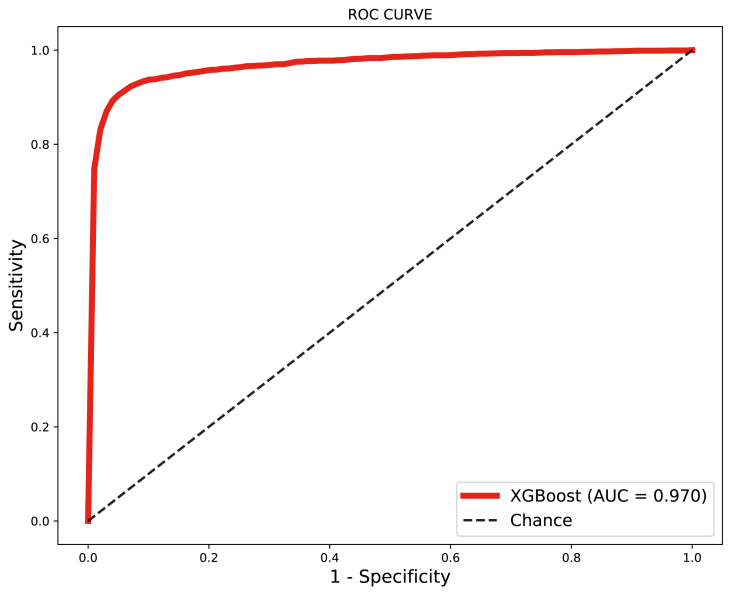
Results using S1-SPNeoDeath as training samples and Test-SPNeoDeath as test samples.

On this experiment, we trained the XGBoost using **S1-SPNeoDeath** and hyper-parameters obtained by fine-tuning. Then we evaluate it using the rest of the samples in **SPNeoDeath** (1.422.357 negative samples and 2377 positive samples, called **Test-SPNeoDeath**). Results obtained on this experiment are depicted on Fig. 14.

Resulting ROC curve is very similar with results presented on Section 4.2, presenting an AUC of 0.97. True positive rate (samples of death occurrences correctly classified) achieved is 91.5% which shows that classifier is very consistent to distinguish between positive and negative class. Table 7 also presents other performance metrics.

**Table 7:** Performance metrics for XGBoost using S1-SPNeoDeath as training set and Test-SPNeoDeath as test set.

S/N	Algorithm	Accuracy	Sensitivity	Specificity	AUC
1	XGBoost	94%	91%	94%	0.970

#### 4.3.2 Variation #2: unbalanced dataset for training

On this experiment, we did split SPNeoDeath in a proportion of 70/30 (70% of positive samples plus 70% of negative samples for training and 30% of positive samples plus 30% of negative samples for testing). This approach generated a much bigger dataset for training but, consequently, leads to the problem of a heavy unbalanced training dataset (which certainly leads to a classifier overfitted to Alive class).

**Fig. 15:**
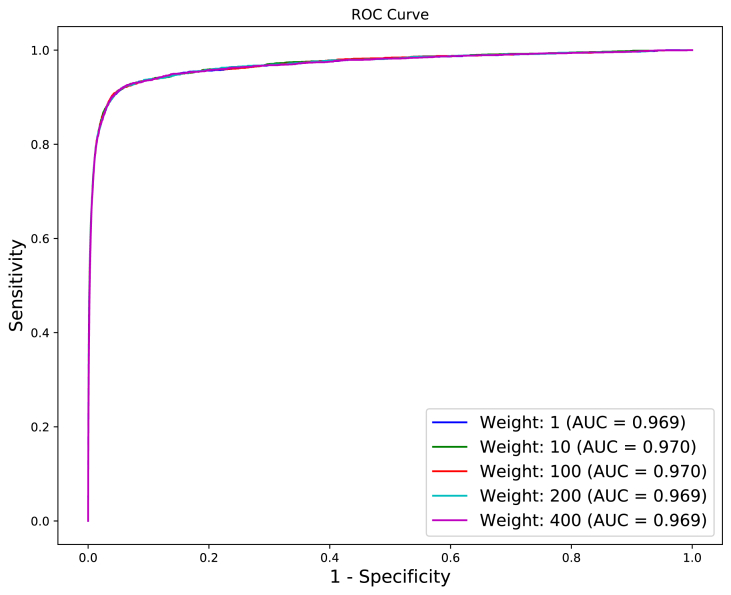
ROC curve using XGBoost and a weight of 400 for positive class in 70/30 validation protocol.

To avoid the overfitting problem as mentioned before, we did use a class weight approach, where the positive class (which has a massive lower number of samples) has its weight increased. This way, when in training process, missclassifications of positive class are much more penalized than missclassifications of negative class. Fig. 15 depicts ROC curves for different values of weights for positive class.

While ROC curves present indistinguishable differences in shapes and AUC, which happens because of the huge difference in the number of test samples in positive and negative class, when analyzing Table 8 we clearly realize the difference that unbalanced dataset causes on Sensitivity and Specificity results.

**Table 8:** Performance metrics for XGBoost classifier with different values to positive class weight.

Positive Class Weight	Accuracy	Sensitivity	Specificity	AUC
1	99%	37%	100%	0.969
10	99%	73%	99%	0.970
100	96%	90%	96%	0.970
200	94%	92%	94%	0.969
**400**	**91%**	**93%**	**92%**	**0.969**

It is also possible to realize that class weight approach compensates unbalanced dataset when using a weight of 400 for positive class, leading to results similar to the ones achieved when training classification method by using a balanced dataset (S1-SPNeoDeath).

From this point into the paper, the rest of the experiments (experiments #3, #4 and #5) have been conducted using 70/30 validation split and a weight of 400.

### 4.4 Experiment #3: model understanding by features importance evaluation

As previously discussed, public health problems demand more than a robust and accurate ML model. They require a model able to explain how to achieve a certain decision (in this case, a low or high probability of death).

**Fig. 16:**
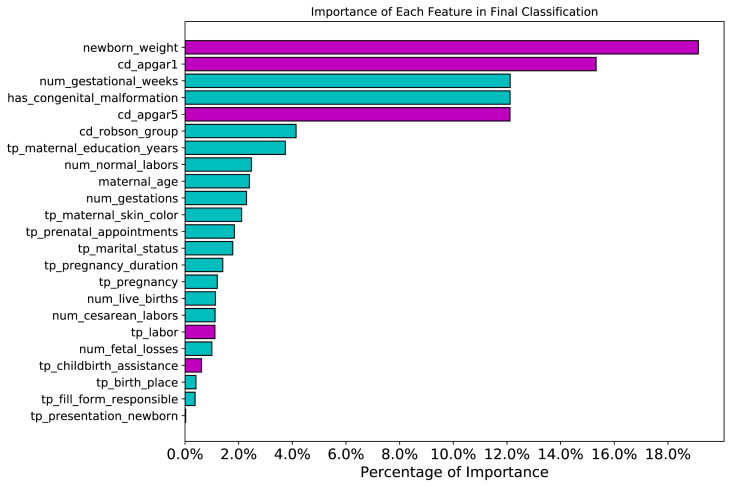
Features importance identified by a combination of XGBoost and SHAP model. Each feature (position on Y axis) represents the average degree of importance for that feature and its calculated over Test-SPNeoDeath samples. Magenta bars represents pos-partum features while cyan bars represents pre-partum features. (For interpretation of the references to color in this figure legend, the reader is referred to the Web version of this article.)

This experiment exposes how the combination of SHAP model and XGBoost can provide model interpretability. For each on training set (which have been used to train the model) we calculate the amount of importance of that feature for the final answer of the trained model. In the end, an average amount is calculated for each feature. Fig. 16 depicts the graph with average importance of each individual feature.

From Fig. 16, its possible to realize that, in average, features *newborn_weight, cd_apgar1, num_gestational_weeks, has_congenital_malformation* and *cd_apgar5* together are responsible for more than 70% of importance in the classifier decision model (with individual importance corresponding to 19.13%, 15.23%, 12.12% 12.12% and 12.11% respectively). From this set of five features, two of them are *pre-partum* variables (considering that *has_congenital_malformation* represent newborn bad formation and that most of these bad formations occurrences can be detected along prenatal appointments, and that *num_gestational_weeks* represents gestational week just before child-birth) and three of them are *post-partum* variables (that only can be obtained from newborn itself).

Despite some demographics studies pointing toward other features to be very important for mortality risk classification (as mother's color or previous living child's), the proposed data-driven model associates a very low importance to most features [4,7,20,31].

More than a feature importance method for a training process, which allows the proposed method point which are the most important features in general, using SHAP values allows the proposed method to provide features of importance in real time when classifying test samples. Using this information, in a decision support scenario, the doctor can use the proposed method to a better understanding of which factors are more critical and for that specific subject.

### 4.5 Experiment #4: pre-partum vs. pos-partum data-driven models

Inspired by previous features importance analysis, the fourth experiment evaluates the proposed method in two different scenarios: in the first scenario we only use post-partum features (*cd_apgar1, cd_apgar5, newborn_weight, tp_labor, tp_presentation_newborn*, and *tp_childbirth_assistance)* to train the data-driven model while in the second scenario, we exclude all the post-partum features from the model.

As previously mentioned, this experiment also has been conducted using 70/30 split of SPNeoDeath as a validation protocol.

**Fig. 17:**
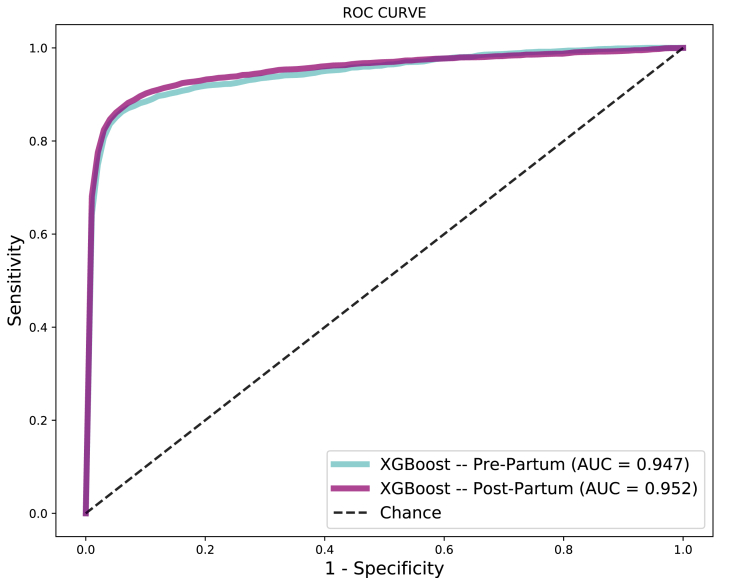
Data-driven models results using pre (cyan) and post (magenta) partum features. (For interpretation of the references to color in this figure legend, the reader is referred to the Web version of this article.)

The major point of evaluating pre-partum and post-partum features separately is evaluating the possibility of achieving a data-driven model able to predict (before labor) the probability of a newborn surviving after 28 days and, in cases where this probability is too low, taking the proper care to avoid newborn death. Fig. 17 depicts the ROC curves for pre-partum and post-partum data driven models. Table 9 presents additional metrics.

**Table 9:** Performance metrics for XGBoost classifier using pre (cyan) and post (magenta) partum features.

S/N	Features	Accuracy	Sensitivity	Specificity	AUC
1	Pospartum	87%	91%	88%	0.952
2	Prepartum	90%	88%	91%	0.947

From ROC curves it is possible to realize that both curves have very similar behavior, with the same AUC of 0.943. When compared against initial classifier trained using all the features, both pre-partum and post-partum classifiers present a ROC curve just 0.02 smaller.

When applying a McNemar's test comparing both classifiers, achieved statistics were 1650.970, and a p-value equal to 0.000, considering 5% of significance, which allows us to claim that, even in conditions where birth weight, 1-min Apgar score and 5-min Apgar score (most representative post-partum features) present normal values, other features as gestational weeks and congenital malformation can lead to an early detection of high death risk. This analysis is better explored on the next experiment.

### 4.6 Experiment #5: pre-partum vs. pos-partum data-driven models: qualitative analysis

As exposed in Section 4.5, pre-partum and post-partum features produce data-driven models statistically different, despite achieving a very similar AUC. In this experiment, we evaluate samples where pre-partum and post-partum models disagree. Specifically, we focus on positive samples (death) that the post-partum classifier missed (predicted wrong answer) but the pre-partum classifier provides the correct answer.

**Fig. 18:**
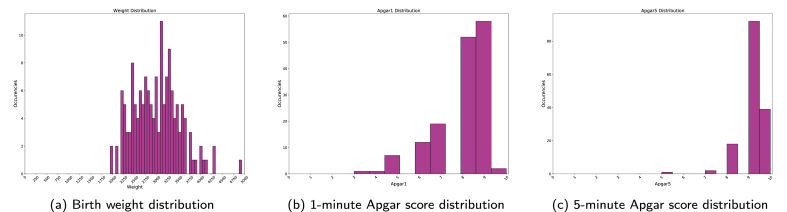
Post-partum most relevant features distributions on misclassified samples.

There were 152 positive samples where the post-partum model constructed on Section 4.5 predicted a wrong answer and the pre-partum model predicted the correct answer. Re-analyzing Fig. 16 and as described on Section 4.4, we can highlight that, birth weight, 1-min Apgar score and 5-min Apgar score are the most relevant features related to post-partum period. Based on both statements, we calculated distribution of these three features from the 152 misclassified samples, as depicted on Fig. 18.

All three distributions are concentrated on regions, where by the current literature, the newborn has good chances to survive (birth weight, 1-min Apgar score and 5-min Apgar score).

**Fig. 19:**
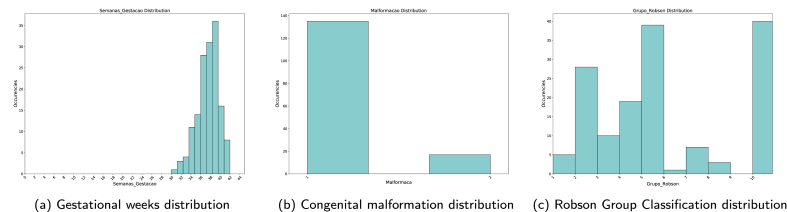
Pre-partum most relevant features distributions on correctly classified samples.

However, in the same samples, distribution of two of the three most important pre-partum features,^2^ as depicted in Fig. 19, already pointed toward high risk of death. Congenital malformation distribution, for example, concentrates most of the occurrences on class 1 (occurrence of malformation). For Robson Group Classification feature, most of the occurrences happen on bins higher equals 4 (higher is worse).

These results point towards the direction that a data-driven model constructed using only pre-partum features can help with newborn mortality risk classification even before birth, which can help pre-care attention programs in cases involving high risk.

## 5 Conclusions and future work

Preventing medical errors is a top priority for any healthcare organization since this can affect the quality of patient care, specially pediatric and neonatal patients which are even more vulnerable to being exposed to errors. Decision-making support tools have a very high potential to address this issue [2].

In this paper we presented a new data-driven method to classify newborns according to the risk of dying until 28 days of life. Composed of three main steps, the proposed method first performs a minor data correction step, to take care of missing and inconsistent values; then, it classifies the sample according to its death risk using a data-driven constructed model based on XGBoost; finally, the model output is explained using SHAP values, providing as final answer the impact of each feature for the final answer of the model.

To evaluate the proposed method, we also proposed a new dataset, named **SPNeoDeath**, comprising more than 1,400,000 samples of birth and deaths from 2012 to 2018 in the city of São Paulo, Brazil.

The Proposed method has been evaluated through five rounds of experiments which evaluated from the most relevant ML method to be used to construct the data-driven model, passing through method effectiveness when applied over more than 1 million samples, features importance for model decision, and finally evaluating method's behavior when trained using just pre or post partum features.

Although many demographic studies point toward high degree of importance for features as race/skin color, marital status and mothers’ years of schooling, data-driven models showed that the most relevant features are related to features birth weight, 1-min Apgar score and 5-min Apgar score, for post-partum features while congenital malformation and gestational weeks for pre-partum features [4,7,20,31].

Achieved results support previous statement that prenatal appointments are very important for child mortality rate reduction, since some very important features, such as congenital malformation, can be detected along prenatal appointments. Even more important, this study points toward the possibility of constructing a data-driven model able to be applied in the real world along the pregnancy period to determine necessary actions to be taken on high death risk cases [23,29].

Pre-partum and post-partum features present results that allow inference in their tarity as shown in qualitative analysis. They can be applied to support decisions even when the odds points toward a not straightforward answer about mortality risk.

For future works, we intend to evaluate the applicability of the proposed model in Brazilian data (not only São Paulo city). Furthermore, we intend to evolve the method to predict not only mortality risk but also mortality rates for specific regions (as cities for example).

## Declaration of competing interest

The authors declare that they have no known competing financial interests or personal relationships that could have appeared to influence the work reported in this paper

## Footnotes

1 In order to allow the reproducibility of the proposed method, along with the comparison with another techniques, source code and dataset are available at https://doi.org/10.7303/syn22240254.

2 Despite some congenital malformation can only be detected after birth, nowadays, most of them can be detected by different exams along appointments during pregnancy.
